# Anti‐spike protein IgG antibody responses up to 3 months after the third dose of the BNT162b2 mRNA vaccine in medical care workers

**DOI:** 10.1002/jgf2.688

**Published:** 2024-03-26

**Authors:** Harukazu Hirano

**Affiliations:** ^1^ Koyo Seikyo Clinic Fukui Health Cooperative Association (FHCA) Fukui Japan

**Keywords:** antibody, COVID‐19, mRNA vaccine, SARS‐CoV‐2

## Abstract

**Background:**

This study aimed to assess the changes in anti‐spike protein IgG antibody titer over time following mRNA vaccination (BNT162b2) against severe acute respiratory syndrome coronavirus 2.

**Methods:**

We monitored IgG levels in 23 medical care workers (MCWs) for up to 3 months after administering the third dose of BNT162b2. Blood samples were periodically collected from all participants.

**Results:**

Following the third dose, the median antibody titer increased to 252 and 327% compared with antibody levels at 1 and 3 months after the second dose, respectively. Additionally, compared with 1 month after the second dose, the median antibody titer decreased to 30.2 and 9.8% at 3 and 6 months, respectively, and to 39.1% at 3 months, compared with 1 month after the third dose.

**Conclusion:**

Antibody levels declined quickly after the second dose but declined more slowly after the third dose, showing a booster effect. This study provides insights into the immunogenicity of booster doses and time intervals for booster vaccination strategies.

## Graphical abstract

In this study, we aimed to assess changes in the anti‐spike protein IgG antibody titers over time following mRNA vaccination (BNT162b2) against severe acute respiratory syndrome coronavirus 2. Antibody levels declined rapidly after the second dose, but declined more slowly after the third dose, showing a booster effect.

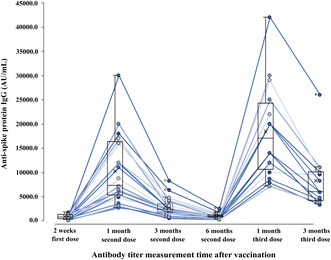

## 1 INTRODUCTION

As of December 2023, the SARS‐CoV‐2 infection, responsible for the COVID‐19 pandemic, has affected 770 million people globally, leading to 7 million deaths.^1^

The BNT162b2 mRNA vaccine, developed to combat the novel severe acute respiratory syndrome coronavirus 2 (SARS‐CoV‐2), has been used globally following its proven efficacy in a randomized controlled trial.^2^ A two‐dose regimen of BNT162b2 was found to be safe, with 95% efficacy, in preventing symptomatic COVID‐19 in persons aged 16 years or older.^2^ In Japan, the vaccination rollout of BNT162b2 commenced on February 17, 2021, initially targeting medical care workers (MCWs); the second dose was administered 3 weeks later. Third dose administration started on December 1, 2021. Regarding the efficacy of the mRNA vaccine, the third additional dose has shown superior humoral immune responses compared with the second dose.^3^, ^4^ A fourth dose was planned with a limited target to prevent aggravation, and its administration began on May 25, 2022. The fifth and sixth doses, whose administering started on September 2022 and May 2023, respectively, are bivalent vaccines against the Omicron variant. The seventh vaccination began in September 2023 as a monovalent vaccine.

The mRNA vaccination program is a novel initiative in Japan, sparking diverse opinions regarding its efficacy and safety among ordinary people as well as doctors. This study was undertaken because of the absence of any published reports on immunoglobulin G (IgG) antibodies after mRNA vaccination in Japan at that time. In addition, this study was initiated concurrently with vaccination work to emphasize *Evidence‐Based Medicine* among our staff in the clinical setting. Considering that some of the staff in my clinic had reservations with regard to vaccination, I was concerned that the vaccination process would not proceed smoothly.

The plaque reduction neutralization test (PRNT), a virus neutralization test, is the gold standard for evaluating vaccine efficacy. However, because PRNT requires expertise and time, commercial serological assays are commonly used, which are simpler, low‐cost, and more practical. Among the SARS‐CoV‐2 structural proteins, the spike and nucleocapsid proteins are the major immunogens. Since the anti‐spike protein (S1 subunit RBD) IgG antibodies correlate with neutralizing antibodies,^5^, ^6^ they are measured to evaluate humoral immune responses after vaccination.

In studies of humoral immunity based on IgG antibody levels, the timing of antibody level measurement after vaccination is crucial. Numerous studies have documented varying decay dynamics of IgG antibodies following COVID‐19 booster vaccinations.^7^, ^8^ However, the precise nature of this decay remains unclear. Some crucial aspects of antibody research include assessing the immunogenicity of vaccines, determining protective thresholds,^9^ and studying adverse reactions.^10^ Despite these goals, antibody titers diminish naturally over time, with an especially rapid initial decay rate that can introduce interval bias to the measurements. To mitigate such bias, this study employed a more precise timing for blood sample collection than prior research endeavors.

## 2 METHODS

### 2.1 Study participants, ethics statement, and vaccine administration

The BNT162b2 vaccination was planned for MCWs from the Fukui Health Cooperative Association (FHCA) from April to June 2021. This study was reviewed and approved by the FHCA Ethics Committee in February 2021 (approval number F21001). All procedures were in accordance with the Declaration of Helsinki. In this study, 469 MCWs from FHCA were called to participate, 23 of whom applied. The protection of personal information and voluntary participation in the study was explained, and written informed consent was obtained from all study participants.

All MCWs received the BNT162b2 vaccine. The first vaccine was administered between April 4 and June 4, 2021, the second dose after 3 weeks, and the third dose8 months after the first (median: 237 d, interquartile range [IQR]: 234–270 d).

### 2.2 Antibody measurement

**FIGURE 1:**
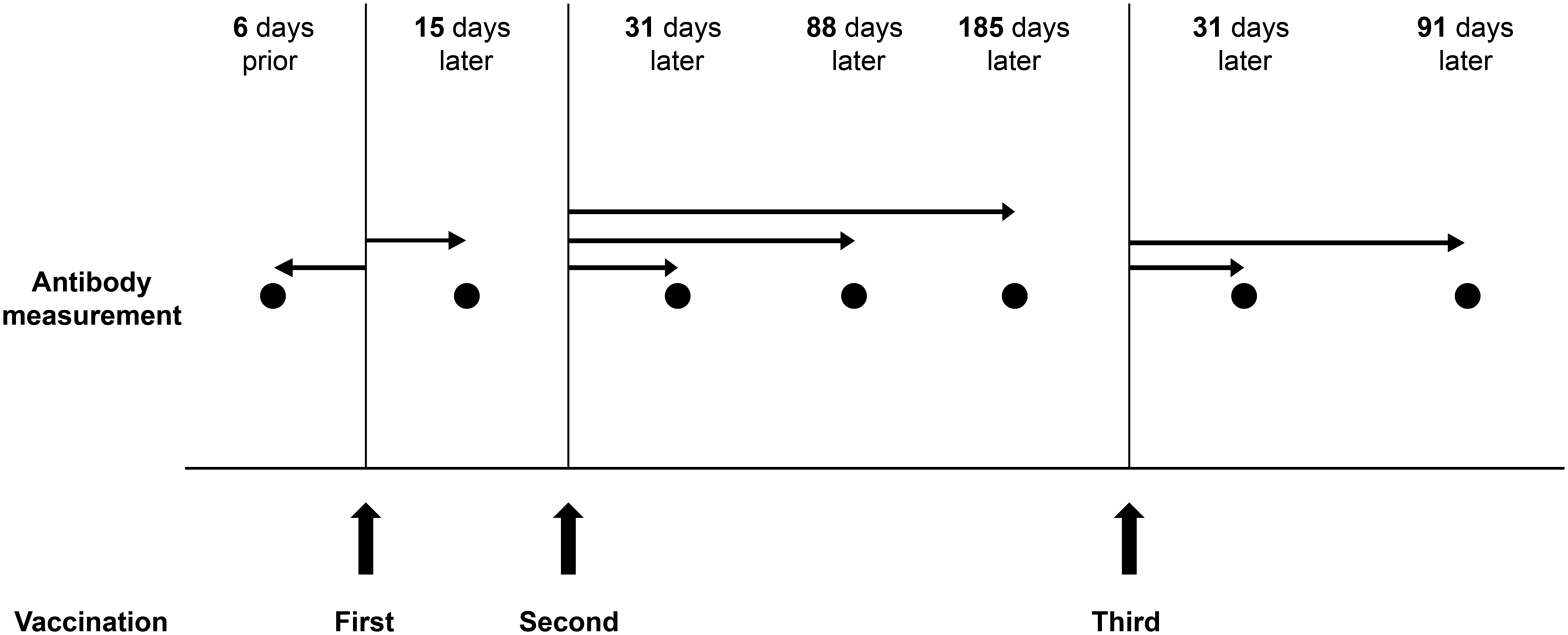
Timeline of vaccination and antibody measurement in MCWs. The number of days between vaccination and antibody measurement is presented as the median.

The ARCHITECT SARS‐CoV‐2 IgG II Quant assay (Abbott) was used for IgG antibody level measurements. This assay is a chemiluminescent microparticle immunoassay (CMIA) used for the qualitative and quantitative determination of IgG antibodies against SARS‐CoV‐2 in human serum and plasma on the ARCHITECT i System. The assay has also been used to evaluate humoral immunity in infected individuals and immunogenicity in individuals who have received mRNA vaccines.^11^ The positive cut‐off antibody level was defined as 50.0 AU/mL as per the manufacturer's instructions. The test sensitivity and specificity were defined at 98.1 and 99.6%, respectively, as per the U.S. Food and Drug Administration.^12^ Blood samples were periodically collected from the participants, and the antibody measurements were outsourced to a commercial laboratory (BML Inc.; Figure 1).

### 2.3 Statistics analysis

The results are presented as the median and interquartile range (IQR) for continuous variables. The median values of the three groups, representing IgG antibody titers after the second dose, were compared using a nonparametric Wilcoxon signed‐rank test. The two groups representing IgG antibody titers after the third dose and groups after the second and third doses were compared using the Wilcoxon rank sum test. The statistical significance level was set at *p* ≤ 0.05.

## 3 RESULTS

In this prospective cohort study, 23 MCWs (8 males, 15 females) were tested for IgG antibodies. The 23 MCWs comprised three physicians, eight nurses, three pharmacists, four care workers, and five medical clerks, with a median age of 55 (IQR: 43–62 years). Of the 23 MCWs, seven had hypertension and one had diabetes, but none were smokers. The median body mass index was 22.8 (IQR: 20.8–25.8). The first test was performed before vaccination (median: 6 d, IQR: 4–9 d), and all participants were confirmed to be negative for antibodies (<50 AU/mL). Table 1 lists the subsequent testing periods. All 23 MCWs participated for up to 6 months after the second dose. However, five participants dropped out of the study 1 month after the third dose, and another four dropped out 3 months after the third dose.

**TABLE 1:** Number of participating MCWs.

	Number of MCWs	Median time after dose (days)	Time range (days) after dose (IQR)	Median antibody levels (AU/mL)	IQR of antibody levels (AU/mL)
2 weeks after first dose	23	15	14–17	622	283–1162
1 month after second dose	23	31	28–33	7144	5181–16,250
3 months after second dose	23	88	85–93	2154	1665–4025
6 months after second dose	23	185	180–193	701	609–1114
1 month after third dose	18	31	24–32	18,000	12,000–25,000
3 months after third dose	14	91	90–92	7040	4152–10,250

Abbreviations: IQR, interquartile range; MCWs, medical care workers.

During the study period, 13 MCWs (57%) underwent polymerase chain reaction (PCR) testing after exposure to a patient with coronavirus disease 2019 (COVID‐19). None of the MCWs had a positive PCR test nor exhibited an increased antibody titer. This result indicates that the MCWs were not infected with COVID‐19. However, anti‐nucleocapsid antibodies were not measured.

Table 1 shows the number of participating MCWs, the period after the first, second, and third doses, and the antibody levels. Only small data scattering was observed between vaccination and antibody testing.

The median antibody titer after the second dose was 7144 AU/mL (IQR:5181–16,250) after 1 month, 2154 AU/mL (IQR:1665–4025) after 3 months, and 701 AU/mL (IQR:609–1114) after 6 months compared with the antibody titer 1 week before administration of the vaccine, which was 622 AU/mL (IQR:283–1162; *p* < 0.0001). Compared with 1 month after the second dose, the median antibody titer at 3 and 6 months decreased to 30.2% (*p* < 0.0001) and 9.8% (*p* < 0.0001), respectively. In addition, there was no significant difference in the antibody titer 6 months after the second dose compared with the antibody titer 1 week before administration of that dose (*p* = 0.11).

**FIGURE 2:**
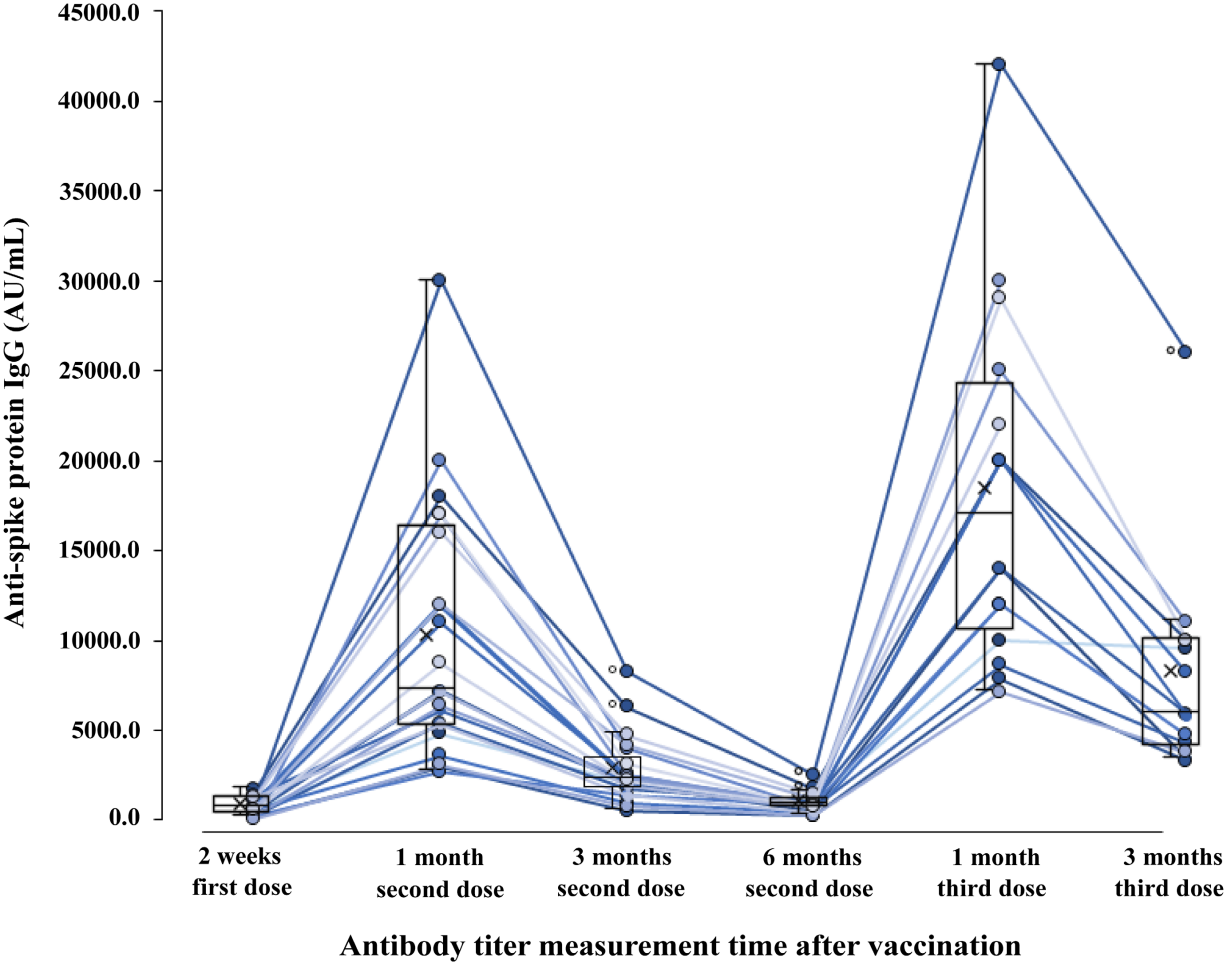
Dynamics in antibody titers up to 3 months after the third vaccine dose. Antibody titers of 23 healthcare workers up to 3 months after the third BNT162b2 vaccination are shown in box plots. AU, antibody units.

The median antibody titer after the third dose was 18,000 AU/mL (IQR:12000–25,000) after 1 month and 7040 AU/mL (IQR:4152–10,250) after 3 months. In addition, compared with 1 month after the third dose, the median antibody titer at 3 months decreased to 39.1% (*p* < 0.01). Compared with one and 3 months after the second dose, the median antibody titer increased to 252% (*p* < 0.01) and 327% (*p* < 0.001) at 1 and 3 months after the third dose, respectively (Figure 2).

## 4 DISCUSSION

Vaccines represent the most effective weapon to control the COVID‐19 pandemic, and monitoring their effectiveness is critical for assessing individual protection against SARS‐CoV‐2 infection. Vaccine protection against SARS‐CoV‐2 infection wanes over time, necessitating the administration of updated booster doses. The effectiveness of booster vaccination depends on the optimal vaccine dose, timing of vaccination, and the pattern of antibody response decay. Additionally, as the type of vaccine, sample collection interval, and type of assay vary from study to study, actual data cannot be easily compared. Table 2 shows several studies that have used methods similar to those in this study. The nine prospective studies listed all targeted MCWs and had relatively small sample sizes with a median of 30 (IQR: 10.5–108). Research with a large sample size was difficult to conduct because the novel coronavirus continued to spread in 2021, and the medical field was under pressure, making it difficult set aside sufficient research capacity.

**TABLE 2:** List of studies on anti‐spike protein IgG antibody after BNT162b2 mRNA second vaccination.

Reference number	Number of participants	Assay manufacturer (units)	Before second vaccination	Time periods from second vaccination to IgG antibody measurement and antibody titer (median; AU/mL BAU/mL)									
				3 days	1 week	2 weeks	3 weeks	4 weeks	6 weeks	3 months	5 months	6 months	9 months
Our study	23	Abbott (AU)	622					7144		2154		701	
18	21	Abbott (AU)	1233		20,227	20,751	15,408						
19	30	Abbott (BAU)		250.8	3491.7	3229.3							
12	86	Abbott (AU)			22,266			9682		2554	1410		
6	22*	Abbott (AU)						13,883				1044	
6	23**	Abbott (AU)						10,736				770	
7	92	Abbott (AU)						8386		2582		870	
13	123	Abbott (AU)			24,534				12,752	5226		1383	
14	158	Abbott (AU)						17,507				1949	

*: 20–39 years old **: 40–59 years old.

In the present study, compared with 1 month after the second dose, the median antibody titer decreased to 30 and 9.8% at 3 and 6 months, respectively. Other studies indicated a decrease in IgG antibody titer to 26% and 30.8% at 3 months compared to 1 month after the second dose,^8^, ^13^ comparable to the results of the present study. In addition, antibody titer decreased to 7.2%, 7.5%, 10.4%, and 11.1% at 6 months compared with those at 1 month after the second dose (Table 2).^7^, ^8^, ^14^, ^15^ In contrast, 3 months after the second dose, the median antibody titer decreased to 30% compared with 1 month later.

Three months after the third dose, the median antibody titer decreased to 39% compared with 1 month later. In addition, the antibody titer 1 and 3 months after the third dose increased 2.5‐fold and 3.3‐fold, compared with the second dose, respectively. Therefore, the rate of decline in the antibody titer 3 months after the third dose was lower than that after the second dose. The increase in antibody titer after the booster shot and the decrease in decay rate indicates a booster effect.

Although our study was conducted up to 3 months after the third dose, a study of 3972 MCWs found that antibody levels declined more slowly 5 months after the third dose of the BNT162b2 vaccine than after the second dose, and the humoral response persisted for 5 months.^16^ A recent Japanese study has also shown that IgG antibody titers are more persistent after the third dose than after the second dose.^17^ Among the 1,137,804 adults aged ≥60 years who received their third dose in Israel, there was a significant reduction in confirmed SARS‐CoV‐2 infections and severe cases, demonstrating the effectiveness of the booster shot.^18^

Humoral immunity is enhanced by the reactivation of memory B cells by the third antigen stimulation with a vaccine or natural infection.^19^ Although not investigated in our study, few prospective cohort studies have investigated short‐term responses related to peaks in antibody titer after BNT162b2 vaccination using the Abbott assay.^20^, ^21^ These studies indicate that IgG antibody titers reach peak levels after 1–2 weeks after the second dose of mRNA vaccines. However, the short‐term response associated with the peak antibody titer after each booster shot of the mRNA vaccine is poorly understood. The dynamics of IgG antibodies after mRNA vaccination have not been fully elucidated, and no mathematical model has been established to date.

Quantitative antibody testing is essential for understanding humoral immunity in vaccinated individuals and populations; however, many testing methods and products exist. To enable the monitoring of antibodies, compatibility evaluation of 125 testing methods has also been performed by the World Health Organization.^22^ Methods for quantifying SARS‐CoV‐2 antibodies using automatic analysis devices include enzyme‐linked immunosorbent assay, chemiluminescent and electrochemiluminescence immunoassays, and CMIA. CMIA (Abbott assay) with high sensitivity and specificity was used in this study. This assay is also available for research in nonlaboratory, general clinical settings.

IgG antibody titers following mRNA vaccination are influenced by age,^23^, ^24^, ^25^ gender,^24^, ^26^ BMI,^27^ smoking,^26^ history of diabetes,^28^ and hypertension.^28^ Although this study targeted healthy MCWs, seven had hypertension and five were over 65 years of age. These demographic and clinical characteristics should be considered when evaluating antibody titers.

Serious adverse events were uncommon, and there were no safety concerns regarding the third dose of the BNT162b2 vaccine.^29^ Monitoring anti‐S‐RBD IgG levels as a correlate of protection will help answer important questions regarding viral neutralization and immunity against SARS‐CoV‐2 in clinical settings. Understanding the dynamics of anti‐S‐RBD IgG will allow healthcare providers to optimize vaccine programs.

The strength of this prospective study was that blood samples were collected regularly from all participants. The limitation of this study is attributed to the small number of participants, 23 of 469 subjects. This is because of the nature of our facility (FHCA), which comprises several small workplaces focused on medical and nursing care. The lack of prior experience with prospective studies, along with limited publicity, contributed to this limitation. Although the significance of vaccines from the perspective of both efficacy and safety should be considered, this study did not examine safety‐related issues, such as side effects.

## 5 CONCLUSION

Our prospective cohort study of 23 MCWs showed that compared with 1 month after the second dose, the median antibody titer decreased to 30.2% and 9.8% at 3 and 6 months, respectively. After 6 months, the antibody titer returned to the baseline titer that was measured 2 weeks before the second dose. Likewise, the median antibody titer decreased to 39.1% at 3 months, compared with 1 month after the third dose. Compared with 1 and 3 months after the second dose, the median antibody titer increased to 252% and 327% after the third dose, respectively. Antibody levels declined quickly after the second doses but declined more slowly after the third dose, showing a booster effect. This study provides insights into the immunogenicity of booster doses and time intervals for booster vaccination strategies.

## FUNDING INFORMATION

Fukui Federation of Democratic Medical Institutions (FFDMI) provided funding for this study (Subsidy No. 20211).

## CONFLICT OF INTEREST STATEMENT

The author has no conflict of interest to declare.

## ETHICS APPROVAL

This study was approved by the Ethics Committee of the Fukui Health Cooperative Association (Approval number 20211).

## Data Availability

Not applicable.

## References

[1] World Health Organization . Coronavirus (COVID‐19) data. https://www.who.int/ Accessed 10 December 2023.

[2] Polack FP , Thomas SJ , Kitchin N , Absalon J , Gurtman A , Lockhart S , et al. Safety and efficacy of the BNT162b2 mRNA Covid‐19 vaccine. N Engl J Med. 2020;383:2603–2615. 33301246 10.1056/NEJMoa2034577 PMC7745181

[3] Gilboa M , Mandelboim M , Indenbaum V , Lustig Y , Cohen C , Rahav G , et al. Early immunogenicity and safety of the third dose of BNT162b2 messenger RNA coronavirus disease 2019 vaccine among adults older than 60 years: real‐world experience. J Infect Dis. 2020;225:785–792. 10.1093/infdis/jiab584 34850049

[4] Lo Sasso B , Agnello L , Giglio RV , Gambino CM , Ciaccio AM , Vidali M , et al. Longitudinal analysis of anti‐SARS‐CoV‐2 S‐RBD IgG antibodies before and after the third dose of the BNT162b2 vaccine. Sci Rep. 2022;12:8679. 35606426 10.1038/s41598-022-12750-z PMC9126106

[5] Padoan A , Bonfante F , Cosma C , Di Chiara C , Sciacovelli L , Pagliari M , et al. Analytical and clinical performances of a SARS‐CoV‐2 S‐RBD IgG assay: comparison with neutralization titer. Clin Chem Lab Med. 2021;59:1444–1452. 33855843 10.1515/cclm-2021-0313

[6] Jung K , Shin S , Nam M , Hong YJ , Roh EY , Park KU , et al. Performance evaluation of three automated quantitative immunoassays and their correlation with a surrogate virus neutralization test in coronavirus disease 19 patients and pre‐pandemic controls. J Clin Lab Anal. 2021;35:e23921. 34369009 10.1002/jcla.23921 PMC8418513

[7] Ikezaki H , Nomura H , Shimono N . Dynamics of anti‐spike IgG antibody level after the second BNT162b2 COVID‐19 vaccination in health care workers. J Infect Chemother. 2022;28:802–805. 35288023 10.1016/j.jiac.2022.02.024 PMC8901382

[8] Olariu TR , Ursoniu S , Marincu I , Lupu MA . Dynamics of antibody response to BNT162b2 mRNA COVID‐19 vaccine: a 7‐month follow‐up study. Medicina. 2021;57:1330. 34946275 10.3390/medicina57121330 PMC8708569

[9] Meschi S , Matusali G , Colavita F , Lapa D , Bordi L , Puro V , et al. Predicting the protective humoral response to a SARS‐CoV‐2 mRNA vaccine. Clin Chem Lab Med. 2021;59:2010–2018. 34492749 10.1515/cclm-2021-0700

[10] Jubishi D , Okamoto K , Hamada K , Ishii T , Hashimoto H , Shinohara T , et al. The association between adverse reactions and immune response against SARS‐CoV‐2 spike protein after vaccination with BNT162b2 among healthcare workers in a single healthcare system: a prospective observational cohort study. Hum Vaccin Immunother. 2022;18:2048559. 35333697 10.1080/21645515.2022.2048559 PMC9115791

[11] Zhao J , Yuan Q , Wang H , Liu W , Liao X , Su Y , et al. Antibody responses to SARS‐CoV‐2 in patients with novel coronavirus disease 2019. Clin Infect Dis. 2020;71:2027–2034. 32221519 10.1093/cid/ciaa344 PMC7184337

[12] U. S. Food and Drug Administration . EUA authorized serology test performance 2022. 2022 https://www.fda.gov/medical‐devices/coronavirus‐disease‐2019‐covid‐19‐emergency‐use‐authorizations‐medical‐devices/eua‐authorized‐serology‐test‐performance

[13] Khoury J , Najjar‐D R , Hanna A , Jabbour A , Abu Ahmad Y , Saffuri A , et al. COVID‐19 vaccine‐long term immune decline and breakthrough infections. Vaccine. 2021;39:6984–6989. 34763949 10.1016/j.vaccine.2021.10.038 PMC8556595

[14] Naaber P , Tserel L , Kangro K , Sepp E , Jürjenson V , Adamson A , et al. Dynamics of antibody response to BNT162b2 vaccine after six months: a longitudinal prospective study. Lancet Reg Health Eur. 2021;10:100208. 34514454 10.1016/j.lanepe.2021.100208 PMC8418937

[15] Bayart JL , Douxfils J , Gillot C , David C , Mullier F , Elsen M , et al. Waning of IgG, total and neutralizing antibodies 6 months post‐vaccination with bnt162b2 in healthcare workers. Vaccine. 2021;28(9):1092. 10.3390/vaccines9101092 PMC8540417 34696200

[16] Gilboa M , Regev‐Yochay G , Mandelboim M , Indenbaum V , Asraf K , Fluss R , et al. Durability of immune response after COVID‐19 booster vaccination and association with COVID‐19 omicron infection. JAMA Netw Open. 2022;5:e2231778. 36107426 10.1001/jamanetworkopen.2022.31778 PMC9478782

[17] Yamamoto S , Oshiro Y , Inamura N , Nemoto T , Horii K , Okudera K , et al. Durability and determinants of anti‐SARS‐CoV‐2 spike antibodies following the second and third doses of mRNA COVID‐19 vaccine. Clin Microbiol Infect. 2023;29:1201.e1–1201.e5. 10.1016/j.cmi.2023.05.020 PMC10207835 37236545

[18] Bar‐On YM , Goldberg Y , Mandel M , Bodenheimer O , Freedman L , Kalkstein N , et al. Protection of BNT162b2 vaccine booster against Covid‐19 in Israel. N Engl J Med. 2021;385:1393–1400. 34525275 10.1056/NEJMoa2114255 PMC8461568

[19] Mise‐Omata S , Ikeda M , Takeshita M , Uwamino Y , Wakui M , Arai T , et al. Memory B cells and memory T cells induced by SARS‐CoV‐2 booster vaccination or infection show different dynamics and responsiveness to the omicron variant. J Immunol. 2022;209:2104–2113. 36426984 10.4049/jimmunol.2200525

[20] Ontañón J , Blas J , de Cabo C , Santos C , Ruiz‐Escribano E , García A , et al. Influence of past infection with SARS‐CoV‐2 on the response to the BNT162b2 mRNA vaccine in health care workers: kinetics and durability of the humoral immune response. EBioMedicine. 2021;73:103656. 34740112 10.1016/j.ebiom.2021.103656 PMC8556513

[21] Gil‐Manso S , Carbonell D , López‐Fernández L , Miguens I , Alonso R , Buño I , et al. Induction of high levels of specific humoral and cellular responses to SARS‐CoV‐2 after the administration of Covid‐19 mRNA vaccines requires several days. Front Immunol. 2021;12:726960. 34671348 10.3389/fimmu.2021.726960 PMC8521189

[22] Knezevic I , Mattiuzzo G , Page M , Minor P , Griffiths E , Nuebling M , et al. WHO international standard for evaluation of the antibody response to COVID‐19 vaccines: call for urgent action by the scientific community. Lancet Microbe. 2022;3:e235–e240. 34723229 10.1016/S2666-5247(21)00266-4 PMC8547804

[23] Romero‐Pinedo S , Quesada M , Horndler L , Álvarez‐Fernández S , Olmo A , Abia D , et al. Vaccine type‐, age‐ and past infection‐dependence of the humoral response to SARS‐CoV‐2 spike S protein. Front Immunol. 2022;13:809285. 35296086 10.3389/fimmu.2022.809285 PMC8918633

[24] Tober‐Lau P , Schwarz T , Vanshylla K , Hillus D , Gruell H . EICOV/COVIM study group, et al. long‐term immunogenicity of BNT162b2 vaccination in older people and younger health‐care workers. Lancet Respir Med. 2021;9:e104–e105. 34687656 10.1016/S2213-2600(21)00456-2 PMC8528470

[25] Müller L , Andrée M , Moskorz W , Drexler I , Walotka L , Grothmann R , et al. Age‐dependent immune response to the Biontech/Pfizer BNT162b2 coronavirus disease 2019 vaccination. Clin Infect Dis. 2021;73:2065–2072. 33906236 10.1093/cid/ciab381 PMC8135422

[26] Nomura Y , Sawahata M , Nakamura Y , Kurihara M , Koike R , Katsube O , et al. Age and smoking predict antibody titres at 3 months after the second dose of the BNT162b2 COVID‐19. Vaccine. 2021;9:1042. 10.3390/vaccines9091042 PMC8472889 34579279

[27] Frasca D , Reidy L , Cray C , Diaz A , Romero M , Kahl K , et al. Influence of obesity on serum levels of SARS‐CoV‐2‐specific antibodies in COVID‐19 patients. PloS One. 2021;16:e0245424. 33760825 10.1371/journal.pone.0245424 PMC7990309

[28] Mitsunaga T , Ohtaki Y , Seki Y , Yoshioka M , Mori H , Suzuka M , et al. The evaluation of factors affecting antibody response after administration of the BNT162b2 vaccine: a prospective study in Japan. PeerJ. 2021;9:e12316. 34721989 10.7717/peerj.12316 PMC8520395

[29] Munro APS , Feng S , Janani L , Cornelius V , Aley PK , Babbage G , et al. Safety, immunogenicity, and reactogenicity of BNT162b2 and mRNA‐1273 COVID‐19 vaccines given as fourth‐dose boosters following two doses of ChAdOx1 nCoV‐19 or BNT162b2 and a third dose of BNT162b2 (COV‐BOOST): a multicentre, blinded, phase 2, randomised trial. Lancet Infect Dis. 2022;22:1131–1141. 35550261 10.1016/S1473-3099(22)00271-7 PMC9084623

